# Antioxidant Properties of Biosurfactants: Multifunctional Biomolecules with Added Value in Formulation Chemistry

**DOI:** 10.3390/biom15020308

**Published:** 2025-02-19

**Authors:** Matilde Tancredi, Carlo Carandente Coscia, Irene Russo Krauss, Gerardino D’Errico

**Affiliations:** 1 Department of Chemical Sciences, University of Naples Federico II, Complesso Universitario di Monte Sant’Angelo, Via Cintia 4, I-80126 Naples, Italy; matilde.tancredi@unina.it (M.T.); carlo.carandentecoscia@unina.it (C.C.C.); irene.russokrauss@unina.it (I.R.K.); 2 Consorzio Interuniversitario per lo Sviluppo dei Sistemi a Grande Interfase (CSGI), Via della Lastruccia 3, I-50019 Florence, Italy

**Keywords:** biosurfactants, rhamnolipids, DPPH assay, antioxidants, formulations

## Abstract

Biosurfactants, amphiphilic metabolites produced by bacteria and yeasts, fulfill a variety of functions in microbial life. They exhibit a well-recognized multifunctionality, spanning from the reduction in surface tension to specific biological activities, including antimicrobial, antiviral, anti-inflammatory, and anticancer effects. These compounds have the potential to serve as environmentally friendly alternatives to synthetic surfactants in industrial formulations, where they could act as emulsifiers and wetting agents. The exploitation of their full potentiality could be a significant added value. Biosurfactants are often cited as effective antioxidants. However, experimental evidence for their antioxidant activity/capacity is sparse. To shed light on the subject, in this review we collect and critically examine all the available literature data for each of the major classes of microbial biosurfactants: rhamnolipids, mannosylerythritol lipids, sophorolipids, and lipopeptides. Despite the variability arising from the diverse composition and polydispersity of the samples analyzed, along with the variety of testing methodologies, the findings consistently indicate a moderate-to-strong antioxidant capacity. Several hypotheses are advanced about the molecular mechanisms behind this action; however, further studies are needed to gain a molecular understanding. This knowledge would fully define the biological roles of biosurfactants and is a prerequisite for the development of innovative formulations based on the valorization of their antioxidant properties.

## 1. Introduction

Biosurfactants are amphiphilic metabolites produced by microorganisms, such as bacteria or yeasts. Their structure comprises one (or more) hydrophilic moiety (the “head”), which can be of saccharidic or peptidic nature, and one (or more) hydrophobic “tail”, arranged in composite molecular architectures [1]. Compared to synthetic amphiphiles, biosurfactants are characterized by a higher eco-compatibility, biodegradability, and biocompatibility, as well as lower toxicity [2]. For these reasons, and due to their pronounced ability to reduce surface tension and act as emulsifiers, biosurfactants have been proposed as environmentally friendly components that could partially replace conventional synthetic surfactants in a wide range of applications, primarily in the oil and detergent industries, but also in the development of biopesticides, food safety processes, and biomedical drug delivery systems [3,4,5,6,7]. However, the practical application of biosurfactants on an industrial scale has, until recently, been constrained by the limited quantities available and the elevated cost. It now appears that these obstacles are about to be overcome. Indeed, the latest developments in metabolic engineering have enhanced the efficacy and security of production through fermentation with non-pathogenic strains. Economically viable and sustainable manufacturing processes utilizing waste-derived substrates, including waste oils and agricultural residues, facilitate circular economy practices and curtail the environmental impact [8].

The availability of biosurfactants presents a unique opportunity for a comprehensive re-evaluation of formulation technology. In microbial life, biosurfactants fulfill a diverse array of physiological roles, ranging from the emulsification and transport of nutrients to interactions with hosts, quorum sensing, the binding of heavy metals (thereby reducing their toxic effects), and the antagonism of competitors [9]. These multifaceted roles underscore the existence of a multitude of functional properties. Indeed, it is now widely acknowledged that, in addition to the well-documented reduction in surface tension and self-aggregation behavior, which are inherent properties of surfactants, biosurfactants have been reported to possess a range of other activities, including antimicrobial, antiviral, antibiofilm, anti-inflammatory, anti-aging, and anticancer effects [10,11,12]. The valorization of these properties would result in a notable shift in the current formulation design paradigms. To achieve this objective, it is essential to base the process on robust scientific knowledge [13].

In addition to the aforementioned benefits of biosurfactants, their antioxidant capacity has emerged as a recurring claim. However, only a limited number of studies have subjected this capacity to rigorous experimental scrutiny, and only a fraction of these have yielded conclusive results. To contextualize the prevailing perception that biosurfactants are potent antioxidants, in this review we collate and critically examine all the available evidence, with the aim of developing a more robust understanding of their efficacy. Following a concise overview of the methodologies employed for the quantitative assessment of antioxidant activity, we present and discuss the findings reported in the literature for each of the main classes of microbial biosurfactants: rhamnolipids, mannosylerythritol lipids, sophorolipids, and lipopeptides. This presentation is concluded with a discussion of the potential for the use of biosurfactants to obviate or diminish the necessity for the incorporation of supplementary synthetic antioxidants into industrial formulations, thereby further facilitating the transition to the green generation of new products.

## 2. Methods

A systematic and comprehensive literature search was performed using Google Scholar, PubMed, and SciFinder as electronic databases. The terms used as keywords for the search were “antioxidant” + “biosurfactant”/“rhamnolipids”/“sophorolipid”/“mannosylerythritol lipids”/“MELs”/“lipopeptides”/“surfactin” (searched for in the titles and abstracts). No temporal limitation was imposed. The full documents were then read to verify that they met the following criteria:

− Inclusion criteria: studies published in English, including articles and patents, reporting direct assays of antioxidant activity of the four classes of microbial biosurfactants of interest (rhamnolipids, sophorolipids, mannosylerythritol lipids, and lipopeptides).
− Exclusion criteria: newspapers, proceedings, and reviews; papers reporting cellular or biological studies for analysis of antioxidant activity; and papers in which biosurfactants were used as carriers of antioxidant molecules.

Finally, the literature database was further enriched by including references reported in the selected papers.

## 3. Antioxidant Assays: Methods for the Screening of Biosurfactants

The actual efficacy of an antioxidant can be assessed quantitatively using specific assays designed to measure the antioxidant activity/capacity of a substance. The two terms “activity” and “capacity” are often considered interchangeable, but according to some authors, they actually indicate different concepts: activity should refer to the rate of action, while capacity refers to the amount of oxidants neutralized and/or radicals scavenged [14]. In another view, activity refers to a single antioxidant, while capacity refers to a mixture of various antioxidants [15]. In the following, we use the term “capacity” because we are mostly dealing with tests that determine the amount or percentage of neutralized oxidants, and the systems under consideration are often mixtures of different components.

Despite the scientific and technological relevance of the subject, the detection of the antioxidant capacity of chemical compounds, including surfactants and biosurfactants, has not been standardized by any official international organization. In the absence of clear reference points, many different methods have been developed, each targeting specific mechanisms of action, broadly categorized as either electron transfer (ET) or hydrogen atom transfer (HAT) processes. The majority of methods adhere to a similar structure, comprising an oxidant generator initiated by an external agent (such as temperature or metal catalysts), a substrate, and an instrumental measure to evaluate the endpoint (for example, UV spectrophotometry) [16,17,18]. Some tests are well standardized, with similar chemicals and procedures used by all research groups. Some other tests may even appear to have the same name and claim to measure the same properties, but on closer inspection they rely on different reactions, reagents, and procedures, which can lead to some confusion and make it difficult to compare results.

An ET method measures the ability of the species of interest, in our case a biosurfactant, to reduce an oxidant by transferring an electron. A DPPH (2,2′-diphenyl-1-picrylhydrazyl) assay quantifies the ability to neutralize a free radical, with the results typically expressed as the IC_50_ values (i.e., the concentration of the tested agent at which the DPPH level is halved, where I stands for inhibition, sometimes referred to as the EC_50_, the half-maximal effective concentration) or the percent inhibition at specific antioxidant concentrations (e.g., 10 mg/mL). Some assays measure the reduction of Fe^3^⁺ to Fe^2^⁺. A ferric-reducing antioxidant power (FRAP) assay employs potassium hexacyanoferrate as the iron(III) source and TPTZ (2,4,6-tris(2-pyridyl)-*s*-triazine) as the linking ligand to the iron ion, and reports the results as the concentration of FeSO_4_ or Trolox equivalents. Another method is a ferric thiocyanate (FTC) assay, which detects lipid peroxidation by quantifying the hydroperoxides. This method measures the oxidation of Fe^2^⁺ to Fe^3^⁺, which forms a thiocyanate complex. The phosphomolybdenum method is based on the reduction of molybdenum(VI) to molybdenum(V), and calculates the antioxidant capacity, expressed as ascorbic acid equivalents.

An HAT method measures the ability of the species of interest to donate a hydrogen atom to an oxidant. A nitric oxide scavenging assay assesses the neutralization of nitric oxide radicals using the Griess reagent to generate a chromophoric azo-derivative and sodium nitrite as the positive control. The results are reported as the percent inhibition or IC_50_ values. An ABTS (2,2′-azino-bis(3-ethylbenzothiazoline-6-sulfonic acid)) radical cation assay quantifies the reduction in ABTS radicals with the results expressed as the inhibition percentages or IC_50_ values. A β-carotene bleaching assay (BCBA) measures the inhibition of β-carotene discoloration caused by linoleic acid peroxidation in emulsions. The results are expressed as the inhibition ratio, which is calculated by comparing the absorbance reduction in the sample with that in the reference sample over a predetermined time at a controlled temperature [19]. A linoleic acid peroxidation assay (LAPA) evaluates the capacity to inhibit peroxide formation in linoleic acid emulsions. Lipid peroxidation is tracked by thiobarbituric acid reactive substances (TBARSs) like malondialdehyde (MDA), which react with thiobarbituric acid (TBA) to form pink chromophores.

Some HAT methods center on the capacity of the examined agent to scavenge reactive oxygen species (ROS), which are frequently implicated in the oxidative damage of materials (e.g., food), and whose imbalance is a prevalent hallmark of oxidative stress in biological tissues. Several approaches can be used for this purpose. The hydroxyl radical scavenging capacity can be assessed by monitoring the inhibition of deoxyribose degradation by hydroxyl radicals (HO·), with results typically expressed as the percent inhibition or IC_50_ values. The superoxide anion scavenging capacity can be evaluated by monitoring the ability of compounds to neutralize superoxide anions (O_2_^−^·), using ferricytochrome C as the chromophore [20]. A hydrogen peroxide assay measures the antioxidant capacity by quantifying the reduction in H₂O₂, which can be detected by the formation of red ferrithiocyanate complexes, using ascorbic acid and cysteine as the positive controls.

In addition to ET and HAT, a third possible mechanism of antioxidant action based on transition metal chelation has recently gained attention. An iron ion chelation assay evaluates the ability to bind metal ions (e.g., Fe^2^⁺), thereby preventing metal-induced radical formation. The results are expressed as the IC_50_ [18].

As will be discussed in the following sections, different methods may yield disparate conclusions regarding the actual antioxidant capacity of a biosurfactant. This discrepancy could be attributed to the varying mechanisms and specific reactions upon which the methods are based. Additionally, it is not uncommon for the results of the same tests, when conducted by different research groups, to vary significantly. This inconsistency could be attributed to the numerous variables involved, including temperature, light exposure, dissolved oxygen, and tube mixing and/or shaking. Therefore, a critical comparison and integrated analysis of the various results is essential to assess the antioxidant potential of biosurfactants, thereby providing a basis for their rational use.

## 4. Biosurfactant Antioxidant Properties

In this section, we present a comprehensive and updated collection of all the antioxidant activity/capacity values of biosurfactants reported in the literature, organized by molecular class.

### 4.1. Rhamnolipids

**Figure 1:**
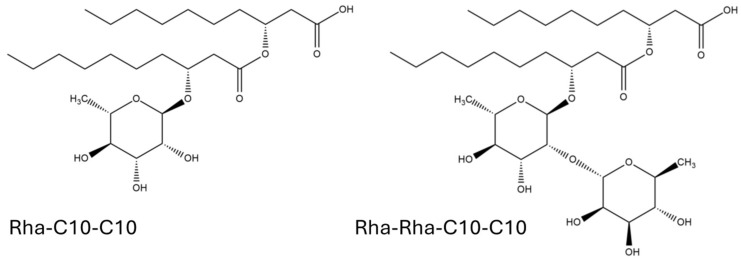
Examples of molecular structures of rhamnolipids tested for antioxidant capacity.

Rhamnolipids are amphiphilic glycolipids with over 60 identified congeners produced by various bacterial species [21]. Their structure consists of a hydrophilic head formed by one or two L-rhamnose units linked by an α-1,2-glycosidic bond, and a hydrophobic region comprising one or two β-hydroxy-fatty acids connected via ester bonds [22]; see Figure 1. These bonds form between the β-hydroxy group of the distal chain and the carboxyl group of the proximal chain, while the carboxyl group on the distal chain typically remains free [23].

Predominantly produced by *Pseudomonas aeruginosa* and other bacteria, but also produced by non-pathogenic fungi [24,25,26] and properly engineered yeasts [27,28], rhamnolipids are thought to perform various functions within bacterial cells. Their functional spectrum likely includes the solubilization of hydrophobic substrates to facilitate their uptake and biodegradation [29], the regulation of the biofilm structure, and the enhancement of bacterial motility. Moreover, they serve as virulence factors by modulating immune responses and disrupting host barriers [30,31]. It is therefore evident that rhamnolipids should be regarded as multifunctional components that play an active role in the complex mechanism that governs microbial life and adaptation.

In addition to their other functionalities, rhamnolipids have recently been identified as compounds with notable antioxidant properties [32,33,34,35,36,37]. This conclusion is based on quantitative analyses carried out on a range of sample types, which revealed their ability to counteract oxidative stress by neutralizing free radicals [34]. These findings suggest that rhamnolipids could represent a sustainable and effective alternative in applications traditionally reliant on synthetic antioxidants. A detailed overview of the results supporting this activity is presented in Table 1. The table summarizes the methods used for production, the assays employed for antioxidant capacity evaluation, and the corresponding results, expressed as the IC_50_ values, inhibition percentages, or reducing capacities.

Overall, the researchers who tested the rhamnolipids concluded that these substances are endowed with antioxidant capacity. However, it is notable that there is a large variability in the reported values. For instance, the range of IC_50_ values reported for the DPPH assays spans from about 77 to more than 2500 µg/mL. This discrepancy may be attributed to the different compositions of the specific samples due to varying culture conditions.

Despite extensive research, no definitive interpretation of the molecular mechanism by which rhamnolipids exert their antioxidant action has yet been proposed. One potential avenue for further investigation is to focus on the saccharidic portion of the molecule. Rhamnose is a 6-deoxyhexose that is relatively uncommon in animals but is frequently found in glycosides derived from plants and bacteria [38,39]. With respect to the majority of the monosaccharide units, which are naturally synthesized as the D-isoform, rhamnose is distinctive in that it predominantly occurs in its L-isoform. As is the case with all monosaccharides, L-rhamnose in its free monomeric form is a reducing sugar, exhibiting a reducing power comparable to that of other aldohexoses [40]. This ability is ascribed to the potential for oxidizing the aldehydic group of the acyclic form to a carboxyl group, and it is expected to be lost when rhamnose is linked to another molecule through a glycosidic bond, thus being constrained in the cyclic pyranose form. Rhamnolipid molecular structures exhibit stable cyclization of the rhamnose units, which would preclude the possibility that they could contribute to the antioxidant activity/capacity of a biosurfactant. However, the presence of rhamnose has been demonstrated to markedly enhance the antioxidant capacity of polysaccharide chains in which the mutarotation process is impeded [41]. It has been proposed that the methyl group present as a side chain plays a significant role, but the precise molecular mechanism remains to be elucidated.

### 4.2. Mannosylerythritol Lipids

**Figure 2:**
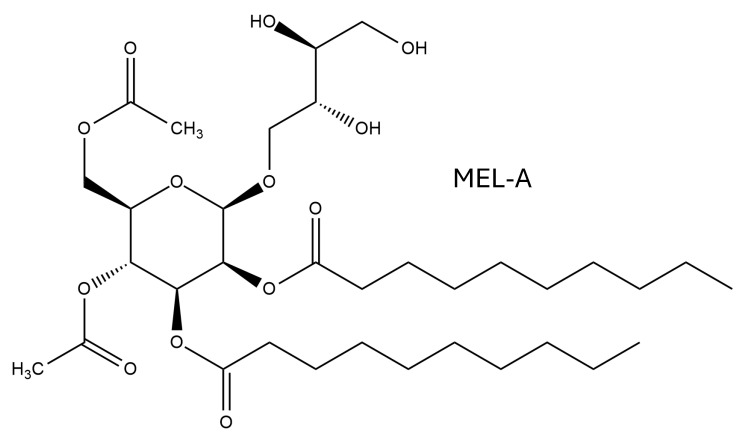
Example of molecular structure of mannosylerythritol lipids tested for antioxidant capacity.

Another class of glycolipid biosurfactants are mannosylerythritol lipids (MELs); in this case, a 4-O-β-d-mannopyranosyl-erythritol moiety represents the hydrophilic headgroup, to which two fatty acids are attached at the C2′ and C3′ positions as hydrophobic tails. This basic structure is shared by four different homologues (from MEL-A to MEL-D), which differ and are classified according to their degree of acetylation: MEL-A is diacetylated at positions C4′ and C6′; MEL-B and MEL-C are monoacetylated at C6′ and C4′, respectively; whereas MEL-D has no acetyl group, as shown in Figure 2. Within each variant of the MELs, there are a variety of fatty acids that differ in the length (C8–C14) and degree of unsaturation, which contribute to the complexity of the congener mixtures obtained by biotechnological approaches [42].

MELs can be produced by different yeasts and fungal strains [43], with *Pseudozyma antarctica* being one of the best producers, using a wide range of raw materials, including agro-industrial waste [44], contributing to their eco-sustainability and making their production advantageous within a bio-circular economy approach [45].

Like other microbial biosurfactants, MELs are characterized not only by impressive surface properties [46], but also by a plethora of biological activities, such as antimicrobial [47], anticancer [48], and anti-inflammatory activities [49]. In particular, MELs are the biosurfactants that have been best acclaimed for their antioxidant properties [50,51,52,53,54], which, together with their ceramide-like features, have led to the development of a flourishing research line dedicated to exploring their potential for use in cosmetic applications, as components of skincare and anti-aging products, for the recovery of skin cells damaged by UV radiation [55], and for the repairing and strengthening of damaged hair [56]. Nevertheless, despite the extensive applicative research conducted in this field, very few articles have reported a quantitative analysis of the antioxidant activity of MELs [57,58], with the quoted results being collected in Table 2.

The reported values indicate that the antioxidant capacity of MELs is contingent upon the microbial strain that produces them. Overall, the authors concluded that MELs have antioxidant capacity. However, no explanation has yet been proposed for the molecular mechanism by which MELs exert this effect. The headgroup of these biosurfactants comprises a mannose unit, for which the same reasoning proposed for the rhamnose in rhamnolipids is applicable. The polyol portion of MELs, erythritol, merits particular attention. This polyol (1,2,3,4-butanetetrol) is naturally found in fruits and fermented foods. Various studies, including *in vivo* investigations, have substantiated the antioxidant properties of erythritol. These findings suggest that erythritol plays a role in mitigating oxidative stress [59,60,61]. Notably, it exhibits the capacity to scavenge hydroxyl radicals. However, it does not react with superoxide radicals, suggesting a selective mode of action [62]. A hypothesis has been advanced proposing a potential pathway through which erythritol operates [59]. This pathway involves the formation of stable byproducts, such as erythrose and erythrulose [59]. Further research is necessary to ascertain whether a similar mechanism could also be operative for MELs.

### 4.3. Sophorolipids

**Figure 3:**
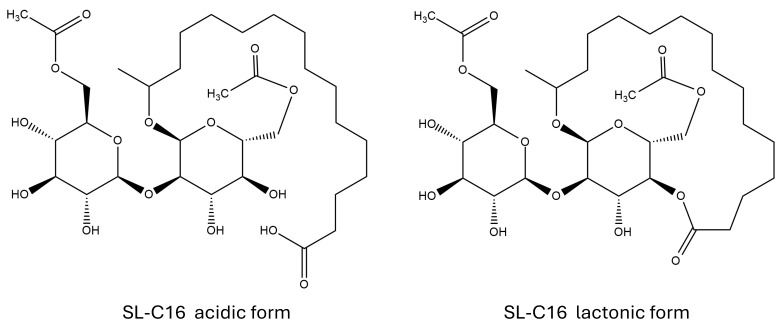
Examples of molecular structures of sophorolipids tested for antioxidant capacity.

Sophorolipids are glycolipids produced by different yeasts as complex mixtures of numerous congeners [63]. These surfactants are composed of a sophorose moiety linked to a long-chain hydroxylated fatty acid through a β-glycosidic bond between the C1′ of the sophorose unit and the ω-1 carbon of the fatty acid [64]. The presence of hydrophilic sugar residues on one side of the long aliphatic chain and the carboxylic group on the other side gives sophorolipids a structure that is similar to that of bolaform surfactants [65,66]. It should be noted, however, that not all carboxylic groups of fatty acids are free. Some may be esterified with the C4″ of the sophorose unit, resulting in the formation of a closed lactonic form of the glycolipid, which is distinct from the open acidic form; see Figure 3. Additional sources of heterogeneity may arise from the potential acetylation of the sugar moiety at the C6′ and/or C6″ position, the length and unsaturation of the hydrophobic tail, and, in the case of the unsaturated forms, from the stereochemistry of the double bond [67].

Sophorolipids offer specific advantages over other microbial biosurfactants. Primarily, they are produced from non-pathogenic yeasts [68], thisallows for a safe industrial production process [69]. Secondly, in regard to their applications, they have been approved with a good safety profile by the Food and Drug Administration (FDA) [70]. The last point, together with their impressive biological properties, including broad-spectrum antibacterial [71], anticancer [72], anti-inflammatory [73], antiviral, and spermicidal activities [74], establishes the basis for their utilization as an additive in food products as well as a potential component of pharmaceutical, cosmetic, and agronomic formulations [68,75]. While there is a substantial body of literature that has investigated the antibacterial and anticancer properties of sophorolipids, there are few reports on their antioxidant capacity [76,77,78,79]; see Table 3.

The reported values point to the significant antioxidant capacity of sophorolipids. It is interesting to note that most of the sophorolipids that have been tested are in the lactone form [76,78,79]. Although there are no studies to prove this point, it is tempting to speculate that this evidence is not coincidental, but may be related to the specific antioxidant activity of the lactone groups [81,82], which is based on the formation of a carbon-centered radical [83]. Also, some antioxidant sesquiterpene compounds are in the lactone form [84,85]. Further research is necessary to investigate this hypothesis in greater depth, to obtain detailed quantitative information, and to gain a mechanistic understanding.

### 4.4. Lipopeptides

**Figure 4:**
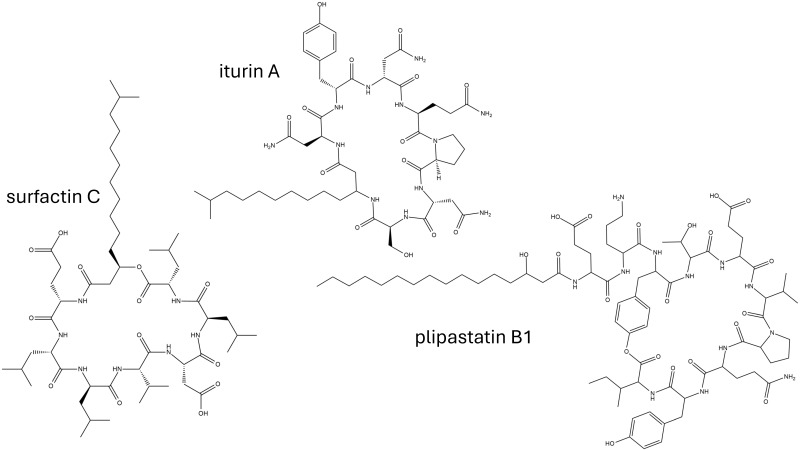
Examples of molecular structures of lipopeptides tested for antioxidant capacity.

Lipopeptides represent a distinctive class of biosurfactants, characterized by a hydrophilic headgroup of peptidic nature, in contrast to the predominantly saccharidic heads observed in the other classes. The common structure of lipopeptides comprises a fatty acid connected to a cyclic peptide moiety [86]; however, the amino acid sequence in the peptide chain and the length and unsaturation degree of the fatty acid residue provide unique properties to each lipopeptide [87]. These biosurfactants are mainly produced by the bacterium *Bacillus subtilis* that secrets three different families of lipopeptides: surfactins, iturins, and fengycins; see Figure 4. Cyclic heptapeptides linked to a β-hydroxy fatty acid with a variable length from C12 to C17 belong to the surfactin family and include surfactins, pumilacidins, and lichenysins. Cyclic heptapeptides linked to a β-amino fatty acid with a variable length from C14 to C19 belong to the iturin family and include iturins A, C, D, and E; mycosubtilins; bacillomycins D, F, and L; and mojavensin. Finally, cyclic decapeptides linked to a C12 to C19 β-hydroxy fatty acid (saturated or not) belong to the fengycin family and include fengycins A and B, plipastatins A and B, and agrastatins [88].

Lipopeptides display additional biological activities besides their typical amphiphilic properties: surfactins exhibit antitumor, antiadhesive, and antimicrobial activities [89]; while fengycins and iturins have mostly been studied for their significant antifungal activity [90,91,92]. In contrast to the extensive research that has been conducted on the potential applications of surfactins, the investigation of these other classes, particularly fengycins, has been significantly less extensive. This is primarily due to the low bacterial productivity observed for these cases [93].

All three families of lipopeptides have been reported to exert significant antioxidant activity, but quantitative investigations are sparse [34,94,95,96,97,98,99,100], as shown in Table 4, and often biosurfactant mixtures have been tested as they are, with no attempt to isolate individual lipopeptide classes or even to determine the mixture composition. In the table, the results for the antioxidant capacity of lipopeptides produced by different microbial strains under different cultivation conditions are reported, including the lipopeptide class and/or the main congener, if reported in the cited reference.

The literature analysis shows that, in some cases, lipopeptides exhibit intrinsic antioxidant capacity [34,98,100], while in other cases, a combination of multiple lipopeptides is required to achieve a significant antioxidant effect [98]. Reframing the problem, there is evidence that the separation of lipopeptides from mixtures is a complex task. In some instances, even the identification of the individual lipopeptides within these mixtures proves to be challenging. For future applications and more precise characterization, it is essential to emphasize that, while lipopeptides have demonstrated antioxidant capacity, further attention should be given to the identification of the specific lipopeptides or combinations of lipopeptides responsible for this activity. Moreover, it cannot be excluded that the presence of certain lipopeptides within a mixture may enhance the antioxidant activity of other lipopeptides in the same mixture.

A closer look at the amino acid composition of lipopeptides could shed some light on the possible role of the peptidic moiety in determining the antioxidant capacities of these biosurfactants. Indeed, the values quoted are of the same order of magnitude as those obtained for some antioxidant peptides [101]. Surfactin has a high content of hydrophobic residues (five out of seven), a feature considered to be a key factor in the ability of peptides to scavenge radicals [102]; in particular, Leu is one of the most abundant amino acids in antioxidant peptides [101]. As for the lipopeptides belonging to the iturins and fengycins family, it is worth noting the presence of Tyr, as the presence of aromatic amino acids is reported to enhance the antioxidant capacity of peptides [103], and Pro, which, with its pyrrolidine ring, could serve as hydrogen donors acting as hydroxyl radical scavengers [101]. Finally, the presence of two Glu residues in the members of the fengycins family may also be important, as negatively charged acidic amino acids exhibit free radical quenching activity due to the presence of excess electrons [104].

However, many caveats are required when analyzing the antioxidant activity of lipopeptides, as the presence of specific amino acids is only one of the factors to be considered, with the relative spatial structure of the amino acids in the peptide sequence also likely to play an important role [105]. Furthermore, different structural features may be more or less important depending on the antioxidant assay used [101]. Thus, this point necessarily deserves further investigation.

## 5. Discussion

The results reported in the previous sections are consistent in showing that biosurfactants can exert some antioxidant activity. However, it is impossible to arrive at single quantitative values describing this effect because of the different composition and polydispersity of the tested samples, which critically depend upon the producing microorganism, the culture medium, and the growth conditions. In most cases, the tests were conducted on biosurfactant mixtures. This observation pertains to all classes of biosurfactants; however, the issue is particularly salient for lipopeptides. Furthermore, the survey has identified additional sources of uncertainty, including the diversity of the tests employed, the variations in the execution of seemingly identical tests by different researchers, and the divergent methods of data presentation. The results are quite scattered and even the computation of the mean values relative to each kind of biosurfactant appears unreliable. As an illustration, the IC_50_ values obtained for the DPPH assays demonstrate a considerable degree of variability for all the considered biosurfactants. The only plausible conclusion that can be drawn is that these values are within a range of 10^2^ to 10^3^ µg/mL for rhamnolipids, sophorolipids, and lipopeptides, while those for mannosylerythritol lipids are an order of magnitude higher (i.e., the antioxidant capacity is lower in the last case).

The molecular mechanisms by which biosurfactants exert their antioxidant action are not yet elucidated. In the previous sections, several hypotheses were advanced. In most cases, a key role seems to be played by the hydrophilic heads, whether saccharide or peptide in nature. The acyl chains, especially if unsaturated, can also play a role. All these hypotheses require further study to confirm or refute. Additionally, the role of self-aggregation leading to micelle/vesicle formation, which could in principle promote cooperative effects, remains to be explored. In summary, further research is needed to determine if, to what extent, and by what mechanism antioxidant activity can be considered among the various biological functions of biosurfactants.

A salient point of discussion pertains to the potential application of this purported antioxidant activity within industrial formulations. Indeed, biosurfactants have predominantly been proposed for at least a partial substitution of synthetic surfactants, particularly due to their higher eco-compatibility, biodegradability, and biocompatibility, as well as lower toxicity [2]. However, it is evident that their full exploitation is contingent upon capitalizing on their multifunctionality. A solid assessment of the antioxidant activity of biosurfactants is the basis for analyzing if they could reduce/avoid the need to include synthetic antioxidants in industrial formulations. These components, of which synthetic phenolic antioxidants (SPAs) are the most widely used, are used in various consumer products, such as food and cosmetics, where they serve to prevent oxidation, thus increasing both stability and shelf life [106]. However, SPAs pose significant health and environmental risks. These petroleum-derived chemicals are synthesized by the catalytic alkylation of phenolic compounds or cresols with olefins [107]. Toxicological research has linked the raw materials used in the synthesis of SPAs to estrogenic, teratogenic, and carcinogenic properties [108]. In addition, environmental challenges have arisen from the persistence of SPAs after wastewater treatment, which leads to the formation of byproducts characterized by increased toxicity [109]. In order to address these limitations, there is a growing trend towards the utilization of natural antioxidants, such as flavonoids, phenolic acids, carotenoids, and tocopherols, as alternatives to synthetic antioxidants [110]. Polymers obtained from natural polyphenols have also shown great promise [111,112]. In light of these findings, it is reasonable to pose the question of whether biosurfactants could also function as antioxidants within chemical formulations.

The most frequently utilized SPAs (e.g., butylated hydroxyanisole, BHA; butylated hydroxytoluene, BHT; and tert-butylhydroquinone, TBHQ) exhibit IC_50_ values for DPPH assays that are at least one order of magnitude lower than those of biosurfactants. This finding indicates that SPAs demonstrate significantly higher efficacy [113,114,115]. Consequently, the elimination of/reduction in SPAs by biosurfactants does not yet appear to be a viable prospect, and further research is required to advance in this direction.

Despite the fact that the present review has not focused on this point, it is interesting, as a final point in this discussion, to mention the fact that biosurfactants are often used not as standalone antioxidants, but as enhancers to boost the antioxidant capabilities of various systems. For instance, mannosylerythritol lipids have been reported to improve the antioxidant efficiency of gold nanoparticles and essential oil emulsions, by stabilizing the active compounds and enhancing their radical scavenging activity [51,116]. Similarly, rhamnolipids are employed as capping and stabilizing agents in the synthesis of zinc oxide nanoparticles, effectively preserving their antioxidant potential over time [117]. Sophorolipids, when combined with essential oils, exhibit a synergistic effect, significantly boosting antioxidant activity [79].

**Figure 5:**
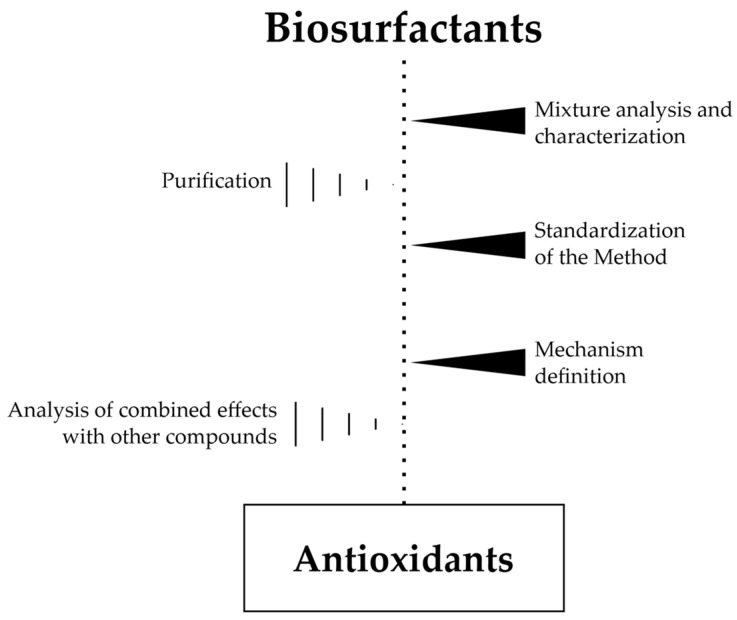
Diagram of scientific progress necessary for the consolidation of the antioxidant properties of biosurfactants.

A schematic representation of the scientific advances needed to place the antioxidant properties of biosurfactants on a solid scientific basis is reported in Figure 5.

## 6. Conclusions

Biosurfactants are substances with a complex molecular architecture that are spontaneously produced by bacteria, yeasts, and other microorganisms to perform multiple biological functions, and therefore have different activities/capacities. Among these, the belief that biosurfactants can exert an effective antioxidant action has gained ground in recent years. The aim of this review was to analyze in detail the scientific basis of this belief.

A careful exploration of the antioxidant capacity values reported in the literature for the various classes of biosurfactants (rhamnolipids, mannosylerythritol lipids, sophorolipids, and lipopeptides) was undertaken. It revealed that, even within a single class, there is considerable heterogeneity in the reported values. This is attributable to a combination of factors, including differences in the composition and polydispersity of the samples tested, as well as the diverse test methods employed. Consequently, quantitative comparisons were deemed to be impractical. In general, the experimental studies have reported fair-to-good (but hardly excellent) antioxidant capacity. In all cases, there is the lack of a clear identification of the molecular mechanisms by which this action is exerted. From this perspective, the present review serves as an opportunity to assess the current landscape and identify avenues for future research. Specifically, there is a need for a more precise and shared standardization of the investigative methods and the direct execution of well-organized comparative studies between different biosurfactants.

Biosurfactants are emerging as suitable components of a new generation of “green” industrial formulations, in which their multifunctionality could be of great added value. From this viewpoint, this analysis of the results from the literature shows that we are still far from being able to prove that the use of biosurfactants could eliminate/reduce the need to introduce antioxidants into these formulations. In conclusion, future studies on the antioxidant activity/capacity of biosurfactants will also be crucial for developing new formulation strategies based on robust scientific knowledge.

## Figures and Tables

**Table 1:** Antioxidant capacity of rhamnolipids produced by various microbial strains under different cultivation conditions.

Microbial Strain	Carbon Source	Predominant Congener	Antioxidant Assay	Antioxidant Capacity	Ref.
*Pseudomonas aeruginosa* DKB1	Bushnell–Haas (BH) mineral medium, + 2% olive oil	Rha-C18	DPPH radical scavenging assay	IC_50_ = 77.18 µg/mL	[32]
			Reducing power assay	IC_50_ = 130.50 µg/mL	
			Hydroxyl radical scavenging assay	IC_50_ = 52.08 µg/mL	
			Nitric oxide scavenging assay	IC_50_ = 95.43 µg/mL	
*Marinobacter litoralis* MB15	Modified Zobel marine broth (MZMB)	Rha-C10-C10	DPPH radical scavenging assay	Inhibition (%) at 5 mg/mL = 72.6%	[33]
*Pseudomonas aeruginosa* MN1	50 g/L of glycerol	Rha-C10-C10	DPPH radical scavenging assay	IC_50_ = 2.28 × 10^3^ µg/mL	[34]
			FRAP assay (ferric-reducing antioxidant power)	Reducing power = 143.2 µM FeSO_4_ at 4.5 mM and 537.2 µM FeSO_4_ at 9 mM	
			Ferric thiocyanate assay (FTC)	IC_50_ = 2.52 × 10^3^ µg/mL	
*Lactobacillus casei subsp. casei* TM1B	Sugarcane molasses	Rha-C16	DPPH radical scavenging assay	IC_50_ = 970 ± 130 µg/mL	[35]
			ABTS radical cation assay	IC_50_ = 600 ± 30 µg/mL	
			Ferrous ion chelating assay	IC_50_ = 790 ± 70 µg/mL	
			Total antioxidant capacity by the phosphomolybdenum method; AAE = ascorbic acid equivalent	57.27 ± 1.23 µg AAE/mg	
*Pseudomonas* MCTG107b	1% w/v glucose	Rha-Rha-C10-C10	DPPH radical scavenging assay	Inhibition (%) at 1 mg/mL = 9.67% ± 3.27	[36]
			ABTS radical cation assay	Inhibition (%) at 1 mg/mL = 14.84% ± 0.4	
*Pseudomonas* MCTG214(3b1)	Not reported	Rha-Rha-C10-C10	DPPH radical scavenging assay	Inhibition (%) at 1 mg/mL = 15.46% ± 4.03	[36]
			ABTS radical cation assay	Inhibition (%) at 1 mg/mL = 10.52% ± 1.75	
*Pseudomonas aeruginosa*	Vegetable oils	Rha-C10-C10	DPPH radical scavenging assay	IC_50_ = 490 ± 80 µg/mL	[37]
			FRAP assay (ferric-reducing antioxidant power)	Trolox equivalents = 0.036 ± 0.002	

**Table 2:** Antioxidant capacity of mannosylerythritol lipids produced by various microbial strains under different cultivation conditions.

Microbial Strain	Carbon Source	Predominant Congener ^a^	Antioxidant Assay	Antioxidant Capacity	Ref.
*Pseudozyma antarctica* T-34	Soybean oil	MEL-A	DPPH radical scavenging assay	Inhibition (%) at 10 mg/mL = 19.6%	[57]
			Superoxide anion scavenging assay	Not statistically relevant	
	Linseed oil	MEL-A	DPPH radical scavenging assay	Inhibition (%) at 10 mg/mL = 43%	
			Superoxide anion scavenging assay	Not statistically relevant	
*Pseudozyma tsukubaensis* NBRC 1940	Olive oil	MEL-B	DPPH radical scavenging assay	Inhibition (%) at 10 mg/mL = 26.4%	[57]
			Superoxide anion scavenging assay	Not statistically relevant	
*Ustilago scitaminea* NBRC 32730	Sucrose	MEL-B	DPPH radical scavenging assay	Inhibition (%) at 10 mg/mL = 16.7%	[57]
			Superoxide anion scavenging assay	Not statistically relevant	
*Pseudozyma graminicola* CBS 10092	Soybean oil	MEL-C	DPPH radical scavenging assay	Inhibition (%) at 10 mg/mL = 16%	[57]
			Superoxide anion scavenging assay	Inhibition (%) at 2 mg/mL = 9.7%	
*Pseudozyma siamensis* CBS 9960	Safflower oil	MEL-C	DPPH radical scavenging assay	Inhibition (%) at 10 mg/mL = 23.3%	[57]
			Superoxide anion scavenging assay	Inhibition (%) at 2 mg/mL = 19.9%	
*Pseudozyma hubeiensis* KM-59	Soybean oil	MEL-C	DPPH radical scavenging assay	Inhibition (%) at 10 mg/mL = 50.3%	[57]
			Superoxide anion scavenging assay	Inhibition (%) at 2 mg/mL = 60%	
*Pseudozyma churashimaensis* OK96	Cuttlefish oil	MEL-A-C16	DPPH radical scavenging assay	IC_50_ = 1.4 × 10⁴ µg/mL	[58]
	Soybean oil	MEL-A-C14:2	DPPH radical scavenging assay	IC_50_ = 2.94 × 10⁴ µg/mL	

^a^ The absence of an indication of the length of the acyl chains indicates that the original reference does not report any information on this point.

**Table 3:** Antioxidant capacity of sophorolipids produced by various microbial strains under different cultivation conditions.

Microbial Strain	Carbon Source	Predominant Congener	Antioxidant Assay	Antioxidant Capacity	Ref.
*Metschnikowia churdharensis f.a.*, sp. Nov, CIG-6A^T^	Glucose 1% (*w*/*v*)	SL-C16; acidic form	DPPH radical scavenging assay	Inhibition (%) at 10 mg/mL = 62.98%	[76]
*Candida bombicola* ACTT22214 ^a^	Glucose 1% (*w*/*v*), molasses 150 g/L, soybean oil 5%	Not specified	DPPH radical scavenging assay	IC_50_ = 6.02 × 10^3^ µg/mL	[77]
*Candida bombicola* ACTT22214 ^a^	Sugarcane molasses and coconut oil	SL-C14; lactonic form, diacetylated	DPPH radical scavenging assay	IC_50_ = 350 µg/mL	[78]
*Starmerella bombicola* (ATCC^®^ 22214™) ^a^	Glucose and oleic acid	SL-C18:1; lactonic form, diacetylated	DPPH radical scavenging assay	Inhibition (%) at 4 mg/mL = 28.31%	[79]

^a^ *Candida bombicola* ACTT22214 and *Starmerella bombicola* (ATCC^®^ 22214™) are the same strain, with the former name being replaced by the latter in recent literature [80].

**Table 4:** Antioxidant capacity of lipopeptides produced by various microbial strains under different cultivation conditions.

Microbial Strain	Culture Broth/ Carbon Source	Predominant Congeners	Antioxidant Assay	Antioxidant Capacity	Ref.
*Bacillus subtilis* SPB1	Mineral salt medium (MSM) + glucose	Mixed lipopeptides	DPPH radical scavenging assay	IC_50_ = 550 µg/mL	[94]
			FRAP assay (ferric-reducing antioxidant power)	2.1 (OD 700 nm) at 10 mg/mL	
			Ferrous ion chelating assay	IC_50_ = 620 µg/mL	
			β-carotene bleaching assay (BCBA)	IC_50_ = 2.26 × 103 µg/mL	
*Bacillus methylotrophicus* DCS1	Luria–Bertani medium and Landy’s medium + glucose, L-glutamic acid, and yeast extract	Mixed lipopeptides	DPPH radical scavenging assay	IC_50_ = 357 µg/mL	[95]
			FRAP assay (ferric-reducing antioxidant power)	3.0 (OD 700 nm) at 2.0 mg/mL	
			Ferrous ion chelating assay	Inhibition (%) at 4 mg/mL = 79.8%	
			β-carotene bleaching assay (BCBA)	IC_50_ = 42 µg/mL	
			Linoleic acid peroxidation assay	Inhibition (%) after 3 days at 0.1 mg/mL = 60.22% Inhibition (%) after 9 days at 0.1 mg/mL = 76.8%	
*Bacillus subtilis* VSG4	Mineral salt medium + glycerol 4% (*v*/*v*)	Mixed lipopeptides	DPPH radical scavenging assay	Inhibition (%) at 5 mg/mL = 69.1%	[96]
			Hydroxyl radical scavenging assay	Inhibition (%) at 5 mg/mL = 62.3%	
*Bacillus licheniformis* VS16	Mineral salt medium (MSM) + glucose	Phospho-lipopeptide biosurfactant	DPPH radical scavenging assay	Inhibition (%) at 5 mg/mL = 73.5%	[96]
			Hydroxyl radical scavenging assay	Inhibition (%) at 5 mg/mL = 68.9%	
*Bacillus amyloliquefaciens* NS6	Minera salt medium (MSM) + yeast extract	Surfactin	DPPH radical scavenging assay	IC_50_ = 1.41 × 103 µg/mL	[34]
			FRAP assay (ferric-reducing antioxidant power)	Reducing power = 255.2 µM FeSO_4_ at 4.5 mM	
			Ferric thiocyanate assay (FTC)	IC_50_ = 1.71 × 103 µg/mL	
*Bacillus mojavensis* I4	Mineral salt medium (MSM) + glucose and glutamic acid	Mixed lipopeptides	DPPH radical scavenging assay	IC_50_ = 360 µg/mL	[97]
			FRAP assay (ferric-reducing antioxidant power)	2.0 (A 700 nm) at 8 mg/mL	
			Ferrous ion chelating assay	IC_50_ = 400 ± 30 µg/mL	
*Bacillus subtilis* SD901	Mineral salt medium (MSM) + Okara (waste of tofu production)	Surfactin [Leu7 or Ile7] (most abundant tail length C14); Surfactin [Val7] (most abundant tail length C14)	DPPH radical scavenging assay	Inhibition (%) is relevant only in mixtures of surfactins/plipastatins at 0.5 mg/mL = 22.88 ± 4.47%	[98]
			Hydroxyl radical scavenging assay	Inhibition (%) = 3%	
			Superoxide anion scavenging assay	Not statistically relevant	
			Hydrogen peroxide assay	Not statistically relevant	
*Bacillus subtilis* LBS1	Luria–Bertani medium and Landy’s medium	Mycosubtilins (most abundant tail length C16)	DPPH radical scavenging assay	Not statistically relevant	[98]
			Hydroxyl radical scavenging assay	Inhibition (%) is relevant only in mixtures of mycosubtilins/surfactins = 15%	
			Superoxide anion scavenging assay	Not statistically relevant	
			Hydrogen peroxide assay	Not statistically relevant	
*Bacillus subtilis* Bs2504	Luria–Bertani medium and Landy’s medium	Plipastatins A and B (most abundant tail length C17)	DPPH radical scavenging assay	Inhibition (%) at 0.5 mg/mL = 18.48 ± 3.83%	[98]
			Hydroxyl radical scavenging assay	Inhibition in both plipastatin and mixtures of plipastatin/surfactin IC_50_ = 222.5 µg/mL	
			Superoxide anion scavenging assay	Inhibition (%) at 0.25 mg/mL = 21% Inhibition (%) in mixtures of plipastatins/surfactins at 0.25 mg/mL = 21%	
			Hydrogen peroxide assay	Inhibition (%) is relevant only for the mixture plipastatins/ surfactins at 0.25 mg/mL = 31%	
*Bacillus subtilis* #309	Sunflower and rapeseed cake medium	Mixed lipopeptides	DPPH radical scavenging assay	Trolox equivalent = 11.67 ± 0.8 µM/g	[99]
			FRAP assay (ferric-reducing antioxidant power)	Trolox equivalent = 0.76 ± 0.096 µM/g	
			ABTS radical cation assay	Trolox equivalent = 3.77 ± 0.22 µM/g	
*Bacillus subtilis* SOPC5	Soybean meal (SBM) and L-glutamic acid	Surfactin	DPPH radical scavenging assay	IC_50_ = 957 µg/mL	[100]

## Data Availability

No new data were created or analyzed in this study. Data sharing is not applicable to this review.
